# De novo CACAN1D Ca^2+^ channelopathies: clinical phenotypes and molecular mechanism

**DOI:** 10.1007/s00424-020-02418-w

**Published:** 2020-06-24

**Authors:** Nadine J. Ortner, Teresa Kaserer, J. Nathan Copeland, Jörg Striessnig

**Affiliations:** 1 Department of Pharmacology and Toxicology, Institute of Pharmacy, Center for Molecular Biosciences Innsbruck, University of Innsbruck, Innsbruck, Austria; 2 Department of Pharmaceutical Chemistry, Institute of Pharmacy, Center for Molecular Biosciences Innsbruck, University of Innsbruck, Innsbruck, Austria; 3 Duke Center for Autism and Brain Development, Duke Child and Family Mental Health and Developmental Neuroscience, Durham, USA

**Keywords:** Voltage-gated Ca^2+^ channels, *CACNA1D*, Neurodevelopmental disorders, Autism spectrum disorders, Calcium channel blockers, Variants

## Abstract

The identification of rare disease-causing variants in humans by large-scale next-generation sequencing (NGS) studies has also provided us with new insights into the pathophysiological role of de novo missense variants in the *CACNA1D* gene that encodes the pore-forming α1-subunit of voltage-gated Cav1.3 L-type Ca^2+^ channels. These *CACNA1D* variants have been identified somatically in aldosterone-producing adenomas as well as germline in patients with neurodevelopmental and in some cases endocrine symptoms. In vitro studies in heterologous expression systems have revealed typical gating changes that indicate enhanced Ca^2+^ influx through Cav1.3 channels as the underlying disease-causing mechanism. Here we summarize the clinical findings of 12 well-characterized individuals with a total of 9 high-risk pathogenic *CACNA1D* variants. Moreover, we propose how information from somatic mutations in aldosterone-producing adenomas could be used to predict the potential pathogenicity of novel germline variants. Since these pathogenic de novo variants can cause a channel-gain-of function, we also discuss the use of L-type Ca^2+^ channel blockers as a potential therapeutic option.

**Electronic supplementary material:**

The online version of this article (10.1007/s00424-020-02418-w) contains supplementary material, which is available to authorized users.

In the past few years the identification of rare disease-causing variants in humans by large-scale next-generation sequencing (NGS) studies has provided us with unprecedented new insights into the physiological and pathophysiological role of ion channels, including voltage-gated Ca^2+^ channels (Cavs, Table 1). The first disease-causing genetic Cav variants were inherited conditions in *CACNA1A* causing familial hemiplegic migraine type 1 and episodic ataxia type 2 [61, 63] or *CACNA1F* causing eye disorders such as congenital stationary night blindness type 2 [84, 86, 96]. The first de novo variants in L-type Cavs were found in *CACNA1C* leading to Timothy syndrome, a multisystem disorder [39, 80, 81]. More recently, high-throughput sequencing of family trios and quads in well-defined disease cohorts in combination with advanced bioinformatic pipelines and the availability of large genetic databases led to the discovery of disease-causing de novo missense variants in the pore-forming α1-subunits of several Cav subtypes (Table 1). This includes variants in *CACNA1E* (Cav2.3 α1) causing developmental epileptic encephalopathies with contractures, macrocephaly, and dyskinesia [22], in *CACNA1G* (Cav3.1 α1) causing childhood-onset cerebellar atrophy [13] and in *CACNA1H* (Cav3.2 α1) causing early-onset hypertension with primary aldosteronism [74]. In addition, there is accumulating evidence that *CACNA1D* (Cav1.3 α1) variants cause an often severe neurodevelopmental syndrome, which is reviewed in this article.

**Table 1:** Voltage-gated Ca^2+^ channels: classification and human genetic diseases

Family		Gene	Protein	Channelopathy
HVA	Cav1	*CACNA1S*	Cav1.1	Hypokalemic periodic paralysis type 1 Malignant hypothermia type 5
		*CACNA1C*	Cav1.2	**Timothy syndrome** Brugada syndrome type 3
		*CACNA1D*	Cav1.3	Sinoatrial node dysfunction and deafness syndrome **Primary aldosteronism** **Neurodevelopmental syndrome with or without endocrine symptoms, autism spectrum disorder**
		*CACNA1F*	Cav1.4	Congenital stationary night blindness type 2 X-linked cone-rod dystrophy type 3
	Cav2	*CACNA1A*	Cav2.1	Spinocerebellar ataxia type 6 Episodic ataxia type 2 ***Familial hemiplegic migraine type 1*** **Congenital ataxia** **Developmental epileptic encephalopathy**
		*CACNA1B*	Cav2.2	n.r.
		*CACNA1E*	Cav2.3	**Developmental epileptic encephalopathy**
LVA	Cav3	*CACNA1G*	Cav3.1	**Childhood cerebellar atrophy** Autosomal dominant cerebellar ataxia Juvenile myoclonus epilepsy
		*CACNA1H*	Cav3.2	Childhood absence epilepsy Autism spectrum disorder **Primary aldosteronism**
		*CACNA1I*	Cav3.3	Schizophrenia risk gene

Human diseases caused by inherited and de novo (highlighted in bold font) missense Cav variants. For familial hemiplegic migraine type 1, both inherited and de novo disease-causing *CACNA1A* variants are described. *HVA*, high-voltage activated; *LVA*, low-voltage activated; *n. r.*, not reported

Interestingly, de novo pathogenic *CACNA1D* variants and similarly those in *CACNA1C*, *CACNA1E*, *CACNA1G*, and *CACNA1H* are not gene-disrupting resulting in a loss of channel function. Instead, they cause typical changes of channel gating, which can enhance channel activity (gain of channel function) during electrical activity patterns in neurons, endocrine, and other electrically excitable cells. These very characteristic gating changes allow the classification of these variants as pathogenic variants in electrophysiological recordings after heterologous expression in mammalian cells (Fig. 1, Table 2). Most variants are located within regions of the pore-forming α1-subunits, which are critical for the function of the activation gate and its control by the channels’ voltage-sensors (see chapter below). Recently published cryo-electron microscopy and crystal structures of two Cav α1-subunits (Cav1.1, Cav3.1; [93, 99]) and bacterial sodium channels (BacNavs) in different states [37, 92] now enable the construction of homology models, which help us to predict how these variants interfere with basic channel functions on the molecular level. Therefore, these variants provide important insight not only into disease but also into the structure-function relationship of Cav α1-subunits.

**Fig. 1:**
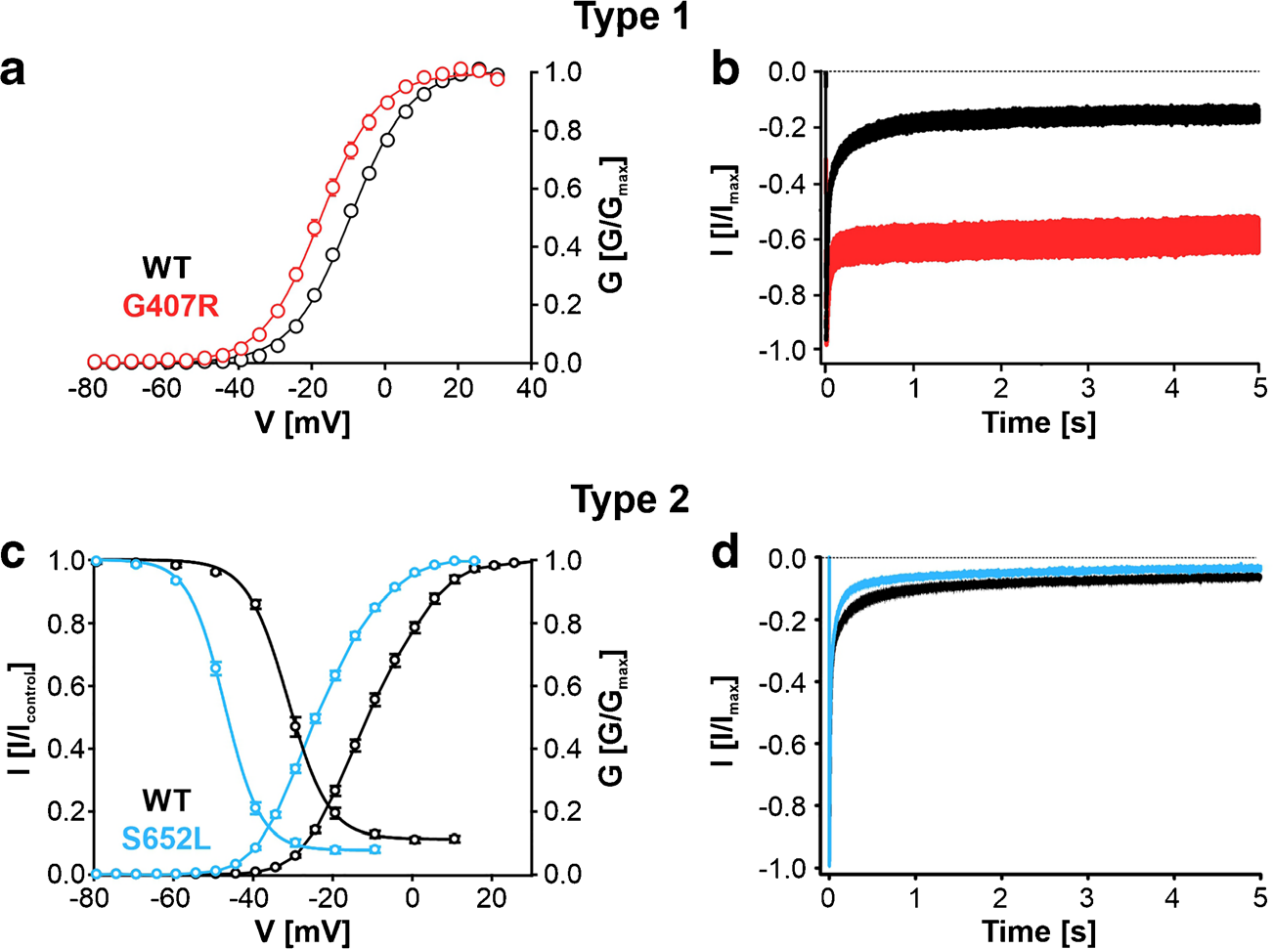
Characteristic gating changes induced by high-risk pathogenic *CACNA1D* variants permitting enhanced channel function of Cav1.3 L-type Ca^2+^ channels (taken and modified from [66, 26]). Typical changes in the voltage dependence of activation (A+C) and inactivation (C) as well as inactivation kinetics during prolonged depolarizations (B+D) are shown for C-terminally short wild-type (black), G407R (red; type 1) and S652L Cav1.3 channels (turquoise; type 2). Note that for the almost non-inactivating G407R variant no steady-state inactivation curve was determined (A). The short Cav1.3 splice variant lacks an intramolecular interaction between the distal and proximal C-terminus which enhances its voltage-sensitivity and inactivation kinetics compared with full-length Cav1.3 channels [8, 77]; however, similar gating changes were also elicited in the long Cav1.3 variant [26, 64]

**Table 2:** Classification of *CACNA1D* missense variants by characteristic functional changes

**Type**	Mutation	Occurrence	Functional changes	ISR sensitivity	References
1	G403D	Germline	**Inactivation almost abolished (voltage-dependence of inactivation not measurable)** Voltage-dependence of activation shifted to hyperpolarized voltages or unchanged		[72]
	G403R	Somatic			[3, 72]
	G407R	Germline			[64, 66]
2	V259D	Somatic	**Voltage-dependence of activation strongly shifted to hyperpolarized voltages** Inactivation not abolished (may be faster, slower, more or less complete after 5 s depolarization to *V*_max_) Voltage-dependence of inactivation strongly shifted to hyperpolarized voltages or unchanged		[3]
	V401L	Germline + somatic		HP -80 mV: enhanced (1.5-fold) HP -50 mV: unchanged	[65]
	S652L	Germline		HP -80 mV: enhanced (3-fold)	[26]
	F747L	Somatic			[66]
	A749G	Somatic			[41, 64, 66]
	I750M	Germline + somatic			[3, 72]
	V1153G	Somatic			[87]
3	Q547H*	Germline	**Slower and less complete inactivation during 3–5 s depolarizations to*V***_**max**_ No change in voltage-dependence of gating		[19]
	P1336R	Somatic			[3]
4	R990H	Somatic	**Mutation-induced (depolarizing) ω-currents**		[52, 66]

Table taken and modified from [66]. Functional changes of Cav1.3 α1 variants were determined upon heterologous expression in mammalian cells (HEK293, tsA201) together with auxiliary β3 (or β1b and β2a in [41]) and α2δ-1 subunits. Isradipine sensitivity was measured using depolarizing standard square pulses to the *V*_max_ (voltage of maximal activation) elicited from a holding potential (HP) of − 50 mV or − 80 mV as indicated. *Q547H: this homozygous variant is not a de novo variant and therefore not further discussed in this review

Gene disrupting de novo variants (frameshift, premature stop codon, splice-donor defect) causing loss of channel function can be reliably predicted using bioinformatic pipelines in most cases. In contrast, it is much more difficult to distinguish high-risk, disease-causing de novo missense variants, for which the functional consequences are difficult to predict in silico, from rare missense variants, which contribute only weakly to disease risk or are even benign. This has important clinical implications, because the symptomatic spectrum of a Ca^2+^ channelopathy should primarily be inferred from rare variants proven to confer high risk in functional studies.

In this review we summarize the spectrum of symptoms associated with a syndrome caused by de novo *CACNA1D* missense variants leading to aberrant gating properties of Cav1.3 Ca^2+^ channels, which support enhanced channel activity especially in cells firing from negative membrane potentials. We update the clinical phenotype of all well-documented pathogenic *CACNA1D* variants affecting a total of 12 individuals. This should help to guide clinical diagnosis and help to outline a rational strategy for potential personalized therapy with Ca^2+^ channel blockers.

## Physiological role of Cav1.3 L-type Ca^2+^ channels

The fact that aberrant gating of Cav1.3 Ca^2+^ channels can cause neurodevelopmental and endocrine symptoms can be explained by the multiple functions of these channels in the mammalian organism. Of the L-type family (Cav1, Table 1), the Cav1.2 and Cav1.3 isoforms show a wide and often overlapping tissue distribution and can be found in most electrically excitable cells (for review see [96]). Cav1.3 channels, although classified as high-voltage activated (HVA, Table 1), can operate at much more negative membrane potentials compared with other HVA Cavs [40, 42], which enables them to support special functions within the auditory, cardiac, endocrine, and nervous system as outlined below. Insight into the physiological roles of Cav1.3 channels came from Cav1.3-knockout mice [54, 67] and humans harboring a mutation in the *CACNA1D* gene resulting in non-functional Cav1.3 channels [4]. In both loss of Cav1.3 function resulted in congenital deafness, bradycardia and sinoatrial node (SAN) arrhythmia (human SAN dysfunction and deafness syndrome, SANDD, OMIM # 614896). The hearing loss can be explained by the important role of presynaptically clustered Cav1.3 channels in cochlear hair cells where they provide Ca^2+^ influx to trigger neurotransmitter release at synaptic ribbons [10, 67, 83]. In contrast, neuronal Cav1.3 channels are predominantly expressed postsynaptically where they shape electrical activity patterns, contribute to dendritic Ca^2+^ signaling, and fine-tune Ca^2+^-dependent gene expression (for review see [96]). In the heart Cav1.3 channels predominate in the SAN and atrio-ventricular node where Cav1.3 Ca^2+^ influx at negative potentials drives the diastolic depolarization required for normal cardiac pacemaking [45, 47, 98], which explains the observed cardiac phenotype in Cav1.3-deficient humans and mice. Cav1.3 also controls endocrine functions in the pancreas and in the adrenal gland. In mice, Cav1.3 does not contribute much to the overall Ca^2+^ current and insulin release from pancreatic β cells [78]. However, in one Cav1.3-knockout mouse line genetic ablation of Cav1.3 induced hypoinsulinemia and impaired glucose tolerance, associated with a deficit in postnatal β cell generation/proliferation ([54], but see [67]). In human pancreatic β cells, Cav1.3 transcripts predominate and seem to be involved in exocytosis [68]. In cultured catecholamine-releasing chromaffin cells of the murine adrenal medulla, Cav1.3 Ca^2+^ channels mediate ~ 25% of the total Ca^2+^ current, support autonomous pacemaker activity [46], and shape secretion-associated firing patterns of these neuroendocrine cells [89]. Cav1.3 is also expressed in aldosterone-secreting zona glomerulosa cells of the adrenal cortex [16, 72]. In these cells, aldosterone synthesis is driven by a periodic intracellular Ca^2+^ signal that mainly depends on low-voltage activated Cav3.2 T-type channels; however, Cav1.3 also contributes to this Ca^2+^ signal (for review see [5]). These special functions of Cav1.3 channels in pancreatic β cells and aldosterone-producing cells nicely explain that individuals harboring an activity-promoting de novo *CACNA1D* variant (germline or somatically in aldosterone-producing adenomas, APAs) can present with primary aldosteronism and/or hyperinsulinemic hypoglycemia (see below, Table 3).

**Table 3:** High-risk disease-causing de novo germline *CACNA1D* variants

Case no.	Variant	Age first symptoms (sex)	ASD	Seizures	Limb spasticity	Hypotonia	Primary aldosteronism	Hypoglycemic hyperinsulinism	Intellectual impairment/disability	Sleep disorder	Autoaggression/self-injury	Developmental delay	Normal MRI	Birth complications	Other	Patho	Ref
1	G403D (ex 8B)	1 month (f)		+	+	+	+		+			+	No	CS (36 weeks of gestation); high birth weight	Sinus bradycardia/biventricular hypertrophy, ventricular septum defect, patent foramen ovale; cortical blindness, cerebral palsy; transient hypoglycemia on day 2	PS2, PS3, PS4, PM1, PM2, PM5	[72]
2	G403D (ex 8B)	Birth (f)	(+)	+	+	+		+	+			+		High birth weight	At age 3 not ambulatory not verbal		[18]
3	I750M	Birth (f)		+	+		+		+	+		+	Yes	CS (41 weeks of gestation), resuscitation	Ventricular hypertrophy; cerebral palsy, spastic quadriplegia, movement disorder with verbal outbursts	PS2, PS3, PS4, PM1, PM2, PM5	[72]
4	V259A	1.5 month (m)		+		+	+		+			+	No	−	Facial dysmorphism microcephaly	PS2, PM1, PM2, PM5	[75]
5	A749G	8 years (f)	+	Normal EEG (year 2)	−	−	−	−	+	+	−	−		CS, floppy infant	Impaired motor skills, clumsy, uncoordinated, night incontinence	PS2, PS3, PM1, PM2	[58]
6	G407R (ex 8A)	15 years (m)	+	−	−	−	−	−	−		−	−	Yes	−	Anxiety, depression;	PS2, PS3, PM1, PM2	[28]
7	V401L (ex 8A)	4 months (m)	+	+	+	+	−	−	+		+	+	yes	−		PS2, PS3, PS4, PM1, PM2, PM5	[65]
8	S652L	Homozygotic twins (m) (current age 13)	+	+ (no recurrence)	−	−	−	−	+		+	+		−	Undescended testes; facial dysmorphism	PS2, PS3, PS4, PM1, PM2	[15]
9	S652L		+	−	−	−	−	−	+		+	+		−	Facial dysmorphism		
10	A749T	1 year (f)	+	−	−	+	−	−	+	+	+	+		−	Binocular vision disorder	PS2, PS4, PM1, PM2, PM5	Unpublished, this review
11	A749T	Details not reported															[82]
12	L271H	Birth (f)		−		+	+	+				+		Born at 32 weeks of gestation	Maternal preeclampsia with HELLP syndrome; facial dysmorphism	PS2, PM1, PM2	[14]

For classifying the pathogenicity (Patho) of the *CACNA1D* variants, we used the criteria proposed in the ACMG classification system [69]. For each of the variants, the combination of the criteria for PS (strong evidence for pathogenicity) and PM (defining moderate evidence for pathogenicity) is given. All variants can be considered “pathogenic” based on ACMG criteria, with the exception of L271H, which is considered “likely pathogenic.” However, as argued in the text, it should be considered a high-risk disease-causing de novo variant. *ASD*, autism spectrum disorder; *CS*, cesarean section; *ex*, exon; *f*, female; *m*, male; +, symptom reported; −, symptom reported to be absent

While the patients with de novo *CACNA1D* missense variants that are described in this review exhibit neurodevelopmental symptoms (Table 3), no brain pathologies have been reported so far for Cav1.3-deficient humans. However, using genetic and pharmacological approaches in mouse models helped to reveal several important functions of Cav1.3 in the central nervous system. Cav1.3 channels account for ~ 10% of total L-type Ca^2+^ channels in the brain and are expressed within multiple regions [6, 78, 79] where they can shape neuronal excitability and induce gene transcription, important for synaptic plasticity, memory formation, and neuronal development. Cav1.3-deficient mice show subtle deficits in proper brain development, evident from a lower number of dopamine-producing neurons in the substantia nigra [62] and a decreased volume and neuron number in the auditory brain stem [24, 25] and the dentate gyrus [48]. The latter was associated with reduced hippocampal neurogenesis and impairments in hippocampus-dependent cognitive functions [48]. Cav1.3 channels are also involved in mood and emotional behaviors. Loss of Cav1.3 resulted in an antidepressant-like phenotype in mice [11], while the opposite was observed upon selective Cav1.3 activation [78]. Also, stimulation of Cav1.3 within the ventral tegmental area (VTA; dopamine midbrain system) was sufficient to elicit the depressive-like behavior together with a social deficit and enhanced cocaine-associated behaviors [49]. This was in line with previous reports linking Cav1.3 activity to the development of psychostimulant-induced sensitized behaviors [20, 33, 71]. An anxiety-like phenotype upon global Cav1.3 knockout was most likely the consequence of the knockout-induced deafness [11], whereas fore-brain specific knockdown of Cav1.3 had no effect on anxiety-related parameters in mice [36]. However, the consolidation of conditioned contextual fear memory is facilitated by Cav1.3 activity, as it was impaired in Cav1.3-deficient mice and associated with significantly reduced long-term potentiation (LTP) in the basolateral amygdala [50, 51]. There is also evidence linking the oscillatory Cav1.3 Ca^2+^ influx in dendrites of autonomously spiking substantia nigra neurons to their selective cell death in Parkinson’s disease ([21] but see [62]).

Given the multiple functions of Cav1.3 Ca^2+^ channels throughout the body, it is plausible that interfering with the way these channels conduct Ca^2+^ ions can result in the dysregulation of several body functions and thus to human diseases.

## Characteristic gating changes of Cav1.3 channels associated with high-risk pathogenic variants

**Fig. 2:**
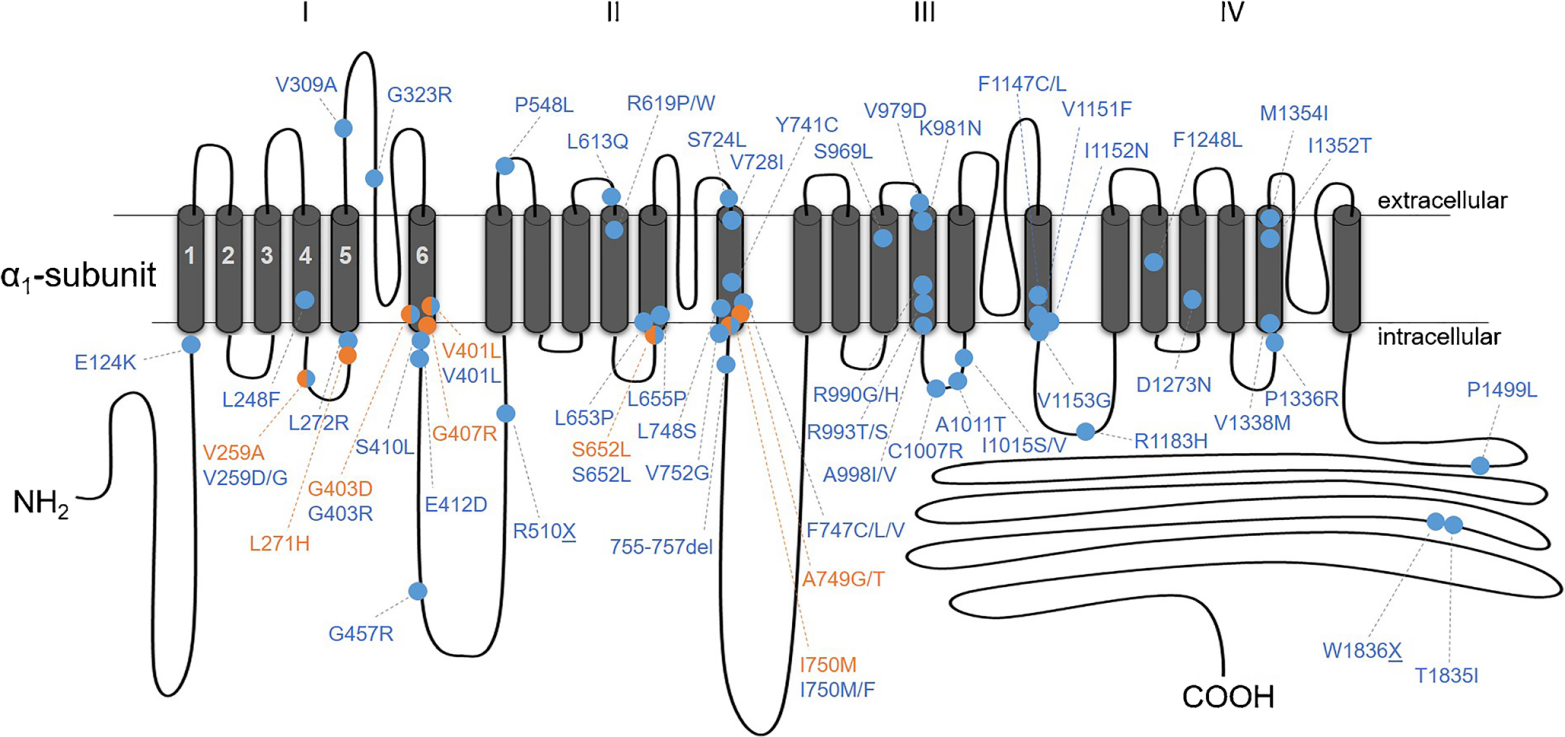
Scheme of the position of de novo germline and de novo somatic APA/APCC variants within the Cav1.3 α1-subunit. The α1-subunit consists of four homologous repeats (I–IV), each comprising six transmembrane segments (S1–S6). From each repeat, S1–S4 form the voltage sensors (S4 contains the positive gating charges) and S5–S6 with their connecting loop build the ion conducting pore. The S4–S5 loop links the voltage-sensor movements to the pore opening. Color code: germline (orange), somatic (blue)

The typical gating changes repeatedly observed in well-characterized patients with a pathogenic *CACNA1D* de novo variant have been described in previous publications (Fig. 1; for references see Table 2). It is important to note that some of these germline variants have also been reported as somatic variants in APAs ([3, 72], Table 2), which likely evolve from aldosterone-producing cell clusters (APCCs) and lesions called APCC-to-APA translational lesions in the adrenal cortex ([2, 9] for review). Since aldosterone production is Ca^2+^-dependent [9], this independently supports the prediction that the observed gating changes cause enhanced Ca^2+^ signaling through the channel. This is also important from a diagnostic standpoint because the pathogenic potential of a newly identified germline *CACNA1D* variant in an affected individual is always unclear, unless in vitro functional data are available. However, if a rare germline variant has also been reported as an APA/APCC variant, for which a much larger number has been identified as compared with germline variants (Fig. 2), this strongly supports its pathogenicity. We have therefore compiled all currently published *CACNA1D* APA/APCCs variants and will discuss them below.

The functional characterization of a larger set of both germline and somatic variants using whole-cell patch-clamp studies by us and others [3, 26, 41, 52, 64–66, 72, 87] now allows to propose at least four characteristic types of functional alterations leading to a channel gain of function. These have been published recently [66] and are depicted in (Table 2).

Type 1 are the inactivation-deficient variants, such as G403D, G403R, and G407R, in which most of the Cav1.3 current fails to inactivate (Fig. 1B). They could also be classified as “Timothy syndrome like” variants because they induce similar gating changes as the Cav1.2 α1-subunit (*CACNA1C*) variants G402S and G406R that affect the corresponding amino acid residue and cause Timothy syndrome, a multisystem disorder also associated with autism [7, 80, 81, 96]. An example is illustrated in Fig. 1A, B for variant G407R (introduced into the short splice-variant of Cav1.3 [66]). Type 2 variants still inactivate to variable extents (i.e., faster or slower than wild-type) but are characterized by pronounced negative shifts of the voltage-dependence of activation with or without a strong negative shift also of the inactivation voltage. An example is shown in Fig. 1C, D for the recently published S652L variant [26]. Type 3 is characterized only by slower and less complete inactivation after 3–5 s at the voltage of maximal activation (*V*_max_), which should favor persistent current in cells during prolonged depolarizations (Table 2). Type 4 are variants in the voltage sensor (e.g., S4 helix positive charges), which enable depolarizing ω-currents as described in several other voltage-gated Ca^2+^ channels [12, 29, 32, 84]. For variants without a functional phenotype in vitro, we cannot exclude pathogenicity through molecular mechanisms which escape our in vitro functional analysis (such as tissue-specific protein-protein interactions disrupted by the variant). However, their role for the disease etiology remains uncertain until further functional or clinical data provide more conclusive insight (e.g., by demonstrating their presence in a larger number of patients and/or in APAs/APCCs).

## Clinical characteristics of germline *CACNA1D* variants

Here we summarize the clinical presentation of 12 individuals with a confirmed or predicted high-risk, pathogenic *CACNA1D* variant for which a well-documented clinical history is available and the “diagnostic” gating changes of Cav1.3 have either been confirmed in functional studies or are strongly supported (variant A749T) by molecular modeling (see below).

The first reported variant (A749G) has been identified in an individual with autism spectrum disorder (ASD) and intellectual impairment but without evidence for additional neurological symptoms ([58, 64]; case no. 5 in Table 3). A subsequent report [72] described pathogenic *CACNA1D* variants in two patients with congenital primary aldosteronism, seizures, and neurological abnormalities (PASNA, OMIM # 615474; cases no. 1, no. 3 in Table 3). However, for both patients it could not be excluded that birth complications contributed to their neurological symptoms. Despite the report of another subject (G407R; case no. 6 in Table 3) affected by ASD without intellectual impairment and neurological symptoms [28, 64], the majority of variants we currently oversee leads to a severe developmental disorder also associated with developmental delay, intellectual impairment, neurological symptoms (including seizures in some of them), and, in several cases, endocrine symptoms, evident as primary aldosteronism, congenital hyperinsulinemic hypoglycemia, or both (Table 3).

Recently, two cases affected by the de novo *CACNA1D* variant A749T were diagnosed (cases no. 10, no. 11 in Table 3). It is located in the same position as the A749G variant described above within the “LAIA” motif discussed in more detail below. Based on its location and the nature of the amino acid exchange, we also predict pathogenicity for this variant, although this needs confirmation in functional studies as for other germline de novo variants (Table 3). Because clinical characteristics have not yet been reported and are also not deposited in online databases, the clinical presentation of subject no. 10 is reported here.

The female patient was referred to a local autism center at 3 years of age. Her medical history revealed diagnoses of ASD, global developmental delay, muscle hypotonia, delayed vision maturation, and difficulty walking. A chromosomal microarray was performed at 20 months which was unremarkable, but a whole exome sequence was completed 2 years later revealing the *CACNA1D* variant. No other genetic abnormalities were found that could explain her symptoms. At the time of presentation to the autism clinic, the patient had been under specialty care from gastroenterology (due to early childhood gastroesophageal reflux and a milk protein allergy), neurology, developmental neurology, developmental pediatrics (medication management of self-harming behaviors), ophthalmology (visual impairment), and orthopedics (for leg/foot misalignment). Despite patient being good-natured and inquisitive, the family was seeking medication management for impulsive and unpredictable self-harming events which occurred multiple times a day including biting herself, scratching to the point of bleeding, throwing herself to the ground, hitting her head against hard surfaces, punching a car so hard it “bloodied her knuckles,” and other significant self-injurious events. Over 2 years of being in the autism clinic, many medications were trialed to provide relief including amantadine, risperidone, memantine, buspirone, quetiapine, gabapentin, and medications targeting underlying anxiety and sleep disorders. Despite periodic improvements with medication management in addition to robust therapeutic services and weekly Applied Behavior Analysis, there was no consistent regimen that controlled her self-harming behaviors and treatment was frequently adjusted. Additionally, a short trial with immediate release isradipine was initiated in an effort to address the underlying calcium channel mutation but was stopped because of concern for worsened symptoms, inefficacy, and complication by a common cold which developed during therapy. During this time, no evidence for cardiovascular abnormalities was found and blood pressure was normal.

From the clinical reports of this and 11 other subjects (Table 3), the following clinically relevant conclusions can be drawn:

1. All affected individuals, except for those with congenital primary aldosteronism (and severe PASNA symptoms, cases no. 1, no. 3, no. 4, and no. 12 in Table 3), have been diagnosed with ASD.
2. Only 2 of the 12 cases (no. 5, no. 6 in Table 3) have been reported with no other neurological or endocrine symptoms in addition to ASD (with or without intellectual impairment) at the time the medical history was published.
3. Endocrine symptoms, driving early genetic diagnosis and immediate therapeutic intervention in the PASNA patients and the individuals affected by congenital hyperinsulinemic hypoglycemia (G403D) or both (L271H, [14]), are observed only in a minority of cases. Their absence does not rule out a *CACNA1D* channelopathy, and their presence cannot be predicted from the variant gating changes: (i) even variants (such as A749G or S652L) causing almost identical biophysical changes as PASNA variants (I750M) [3, 26, 64, 66, 72] were not associated with clinically overt endocrine symptoms; (ii) an identical variant (G403D) may cause severe disease but with different endocrine manifestations (primary aldosteronism in case no. 1, congenital hyperinsulinemic hypoglycemia in case no. 2); (iii) endocrine symptoms may resolve with age [18, 35].
4. Although endocrine symptoms are not consistently part of the syndrome, individuals with all pathogenic *CACNA1D* variants should be monitored for symptoms of primary aldosteronism (see case no. 12, [14]) and altered glucose homeostasis even if not present in the peri- and postnatal period. Cav1.3 channels also contribute to the electrical activity and adrenaline secretion in adrenal chromaffin cells in rodents. At present it is unclear if this also results in abnormal adrenaline release in humans. However, measurement of plasma catecholamine levels in affected individuals could address this important question, considering that anxiety is often reported in these patients.
5. Facial dysmorphism or syndactyly, as described in subjects with *CACNA1C* gain of function variants ([96], see also review in this issue), is not a consistent finding in the *CACNA1D* cohort (Table 3) but can serve as a supporting diagnostic feature.
6. Autoaggressive and self-harming behaviors have been well-described in the clinical histories of two individuals with different variants (cases no. 7, no. 10) and are considered a behavioral abnormality challenging caregivers and treating physicians.

As described above, Cav1.3 L-type channels are also present in other tissues. Although homozygous loss of Cav1.3 function results in congenital deafness and sinoatrial node dysfunction, it is at present unclear if the heterozygous gain-of-function gating changes of the reported variants result in clinically relevant symptoms on hearing or directly cause the cardiac abnormalities observed in some cases (Table 3).

## Repurposing of Ca^2+^ channel blockers (“Ca^2+^ antagonists”) for symptom control

As outlined above, the heterozygous de novo *CACNA1D* variants are dominant in nature and the disease cannot be explained by heterozygous loss of channel function, which is apparently asymptomatic in mice and humans. This is further supported by at least 10 heterozygous protein loss of function variants (stop gained or frameshift located N-terminal to the beginning of the C-terminus) reported in gnomAD (gnomad.broadinstitute.org) samples so far. Based on the enhanced channel activity in heterologous expression systems and promoted Ca^2+^ signaling in APAs, it is likely that the altered gating changes also increase Cav1.3 channel activity in some populations of neurons. Thus, inhibition of Cav1.3 channel activity with drugs appears as a potential treatment option in affected individuals. Although neurodevelopmental defects are unlikely to be completely reversible, treatment of some otherwise difficult to control symptoms (such as seizures, self-harming behaviors, or muscle hypotonia) may considerably improve the quality of life of patients and their caregivers.

Treatment with Ca^2+^ channel blockers has already been described in some of the patients, but no conclusive results regarding improvement of neurological or neuropsychiatric symptoms have yet been obtained. The clinical course of the patient described in case no. 1 (G403D, PASNA) was notable for uncontrolled hypertension with hypokalemia (due to primary aldosteronism). Treatment with the dihydropyridine (DHP) Ca^2+^ channel blocker amlodipine normalized blood pressure and resolved biventricular hypertrophy. Effects of this treatment on other underlying symptoms were not reported [72]. The patient of case no. 2 (same variant) with congenital hyperinsulinemic hypoglycemia received diazoxide (only required until age of 5 years), which successfully controlled blood glucose. Although considered a therapeutic option, treatment with a Ca^2+^ channel blocker was not reported [18]. As mentioned in the detailed case report above, short treatment of subject no. 10 with the DHP isradipine was also inconclusive. Interestingly, the young subject no. 12 was treated with nifedipine oral solution every 8 h to control hypertension. This not only controlled hypertension but also improved muscle hypotonia. The extent of this improvement was not quantified, and the long-term outcome as well as the tolerability of this treatment was not reported [14]. Nevertheless, it supports the hypothesis that some symptoms may improve upon treatment with DHPs.

What is known about the pharmacology of Cav1.3 that could guide off-label treatment trials in subjects with confirmed pathogenic *CACNA1D* variants? Both pharmacokinetic and pharmacodynamics need to be considered.

The first question is if Ca^2+^ channel blockers can sufficiently engage Cav1.3 Ca^2+^ channels in the brain at plasma levels achieved for antihypertensive therapy. Pharmacokinetic studies in rodents clearly show that some DHPs, such as felodipine and isradipine, used since decades for the treatment of high blood pressure, can quickly and efficiently cross the blood-brain barrier ([76], for review see [43]). An exception seems to be amlodipine. For this widely used DHP, brain exposure after a single dose seems to be lower [88]. Since this compound’s long half-life requires many days of dosing to reach steady state, it is unclear if brain exposure can further increase after multiple dosing.

The second question is if Cav1.3 channels in the brain are efficiently blocked at therapeutic doses of DHPs. The therapeutic target of DHPs for cardiovascular indications is Cav1.2 L-type channels in arterial smooth muscle cells [78, 85]. It is known that under identical experimental conditions Cav1.2 channels are about 5 times more sensitive to inhibition by DHPs than Cav1.3 [62, 94]. This means that higher doses may be required to inhibit Cav1.3 channels in the brain compared with Cav1.2 in the periphery [62]. However, we have recently discovered that some variants can enhance the sensitivity of Cav1.3 channels for inhibition by the DHP isradipine, as shown for S652L [26] and, to a smaller extent, for V401L [65]. This has meanwhile been confirmed also for other variants (NJO, unpublished data). However, we also found the opposite, variants which significantly reduce sensitivity to isradipine (unpublished). This strongly suggests that therapeutic trials should be first started in subjects with DHP-sensitizing variants. If therapy fails in these patients it is unlikely that subjects with other variants would benefit from DHP therapy.

In addition, other factors could be therapy limiting: DHPs are very well tolerated by most patients but may cause hypotension and dizziness at higher doses. Immediate release preparations should be avoided because fast blood pressure lowering may induce sympathetic activation, reflex tachycardia, and flushing, which may cause unwanted behavioral reactions in some patients. Therefore, suitable DHPs require extended release formulations to prevent fast onset, which can be a problem in patients with feeding problems or younger patients, in which oral solutions are more appropriate for administration and correct dosing. In addition, DHPs are cytochrome-P450-3A4 substrates and drug-drug interactions with concomitant therapies (e.g., antiepileptic or psychiatric drugs) have to be considered.

Nevertheless, despite many open questions and potentially therapy-limiting considerations, treatment with Ca^2+^ channel blockers remains a therapeutic option that should be explored carefully in these patients. These experimental therapies will require not only a skilled therapist but also the patience and support of cooperative parents and their qualified assessment of predefined treatment outcomes.

## Somatic *CACNA1D* APA/APCC variants can help to classify germline variants as high risk

As outlined above, somatic *CACNA1D* variants in APAs and APCCs cause excess aldosterone production and primary aldosteronism although they seem not to directly contribute to abnormal cell proliferation and adenoma formation [97]. This finding strongly suggests that the gating changes induced by these variants indeed permit enhanced Ca^2+^ signaling through Cav1.3 channels in these human tumor cells. Accordingly, several variants found in APAs were also reported as pathogenic germline variants (Fig. 2) in a subject with (I750M) and others without endocrine symptoms (S652L, V401L). Therefore, APA/APCC variants could guide the assessment of the potential pathogenicity of new germline variants for which no functional data are available and thus could aid clinical diagnosis. However, a small percentage of APAs and APCCs cannot yet be explained by known somatic de novo mutations [9]. Therefore, it cannot be ruled out completely that a *CACNA1D* variant is in fact benign and other yet unknown genetic factors account for excess aldosterone production in a given APA/APCC. We therefore propose criteria which classify these somatic variants as pathogenic, likely pathogenic, likely benign or of (yet) uncertain pathogenicity (Table 4). The classified somatic APA/APCC variants as well as their approximate position within the Cav1.3 α1-subunit are given in Table 5 and Supplementary Figure 1, respectively.

**Table 4:** Prediction of pathogenicity in novel germline *CACNA1D* variants using information from somatic mutations in APAs and APCCs

Classification	Criteria
Pathogenic	If absent in controls and typical gating changes (Table 2) have been observed in functional studies
	If absent in controls and has been described independently in at least **two** different adrenals
	If present in a single control, described independently in at least **two** different adrenals and typical gating changes (Table 2) have been observed in functional studies
	If absent in controls and has been described in only one adrenal but at least one additional variant (pathogenic/likely pathogenic) is described in the same position
Likely pathogenic	If present in a single control and described independently in at least **two** different adrenals
	If absent in controls and has been described in only one adrenal
Likely benign	If present in more than 3 controls and only one adrenal
	If the variant likely represents a null variant (loss of channel function, nonsense, frameshift, canonical ± 1 or 2 splice sites, initiation codon, single or multiexon deletion)
Uncertain	If present in one control and described in only one adrenal
	If present in more than one control and described in more than one adrenal

The criteria listed in the table were used to classify the pathogenicity of somatic mutations in APAs and APCCs assuming that some of them cannot (yet) be considered pathogenic mutations causing high risk for disease based e.g. on the frequency of them being independently reported and their occurrence in healthy control individuals. Note that this classification should aid in predicting the potential pathogenicity of germline mutations for endocrine or neurodevelopmental syndromes associated with *CACNA1D* variants. In the absence of a suitable guideline for classifying the potential pathogenicity for somatic tumor mutations [38], we consider the here described criteria for classification of the pathogenicity of *CACNA1D* APA/APCC variants

**Table 5:** Predicted pathogenicity of somatic *CACNA1D* variants reported in APAs and APCCs

Position	Variant	Reference	Gating change	Times reported	Reported in gnomAD (#)	Pathogenicity
E124	E124K	[60]	-	1	-	Likely pathogenic
	Other gnomAD entries at this position:				E124Q (1×) E124D (3×)	
L248	L248F	[60]	-	1	-	Likely pathogenic
V259	V259A	[75]	-	1× (germline)	-	
	V259D	[3, 17]	Type-2 [3]	3	-	Pathogenic
	V259G	[60]	-	1	-	Pathogenic
L272	L272R	[60]		1	-	Likely pathogenic
V309	V309A	[56]	-	1	-	Likely pathogenic
	Other gnomAD entries at this position:				V309I (84×; HOM: 1×)	
G323	G323R	[60]	-	1	-	Likely pathogenic
V401	V401L	[65]	Type-2 [65]	1× (germline)	-	
	V401L	[1, 56, 60]	Type-2 [65]	4 (exon 8a)	-	Pathogenic
G403	G403D	[18, 72]	Type-1 [72]	2× (germline)	-	
	G403R	[1, 3, 17, 34, 56, 57, 59, 60, 72, 73, 95, 100]	Type-1 [3,72]	54 (exon 8a: 32; 8b: 12; ns: 10)	-	Pathogenic
	Other gnomAD entries at this position:				G403dup (2×) loss of function, exon 8b [4]	
S410	S410L	[59, 60]	-	2	-	Pathogenic
E412	E412D	[95]	-	1	-	Likely pathogenic
G457	G457R	[59]	-	1	1×	Uncertain
	Other gnomAD entries at this position:				G457del (2×)	
R510 (R530)	R510X	[59]	-	1	-	Likely benign
	Other gnomAD entries at this position:				R510Q (2×)	
P548 (P568)	P548L	[59]	-	1	-	Likely pathogenic
L613 (L633)	L613Q	[57]	-	1	-	Likely pathogenic
R619 (R639)	R619P	[56]	-	1	-	Likely pathogenic
	R619W	[57]	-	1	1×	Uncertain
S652 (S672)	S652L	[15]		2× (germline)*	-	
	S652L	[17, 56, 60, 95]	Type-2 [26]	5	-	Pathogenic
	Other gnomAD entries at this position:				S652W (3×)—no gating change [26]	
L653 (L673)	L653P	[60]	-	1	-	Likely pathogenic
L655 (L675)	L655P	[17]	-	1	-	Likely pathogenic
	Other gnomAD entries at this position:				L655F (1×)	
S724 (S744)	S724L	[60]	-	1	-	Likely pathogenic
V728 (V748)	V728I	[90]	-	1	115×	Likely benign
Y741 (Y761)	Y741C	[17]	-	1	-	Likely pathogenic
F747 (F767)	F747C	[55, 56, 59]	-	3	-	Pathogenic
	F747L	[1, 3, 17, 56, 57, 59, 60, 87, 95]	Type-2 [66]	21	-	Pathogenic
	F747V	[17, 55, 57, 59, 60, 73, 95]	-	18	-	Pathogenic
L748 (L768)	L748S	[60]	-	1	-	Likely pathogenic
I750 (I770)	I750M	[72]	Type-2 [72]	1× (germline)	-	
	I750M	[3, 17, 34, 56, 72, 73, 95]	Type-2 [72,3]	11	-	Pathogenic
	I750F	[17, 56]	-	2	-	Pathogenic
V752 (V772)	V752G	[95]	-	1	-	Likely pathogenic
755-757del (775-777del)	Deletion of “LAD’	[60]	-	1	-	Likely pathogenic
	Other gnomAD entries at this position:				A756T (1×)	
S969 (S989)	S969L	[60]	-	1	-	Likely pathogenic
V979 (V999)	V979D	[17]	-	1	-	Likely pathogenic
	Other gnomAD entries at this position:				V979I (1×)	
K981 (K1001)	K981N	[17]	-	1	-	Likely pathogenic
R990 (R1010)	R990G	[56]	-	1	-	Pathogenic
	R990H	[3, 59, 60, 87, 95]	Type-4 [52]	9	-	Pathogenic
R993 (R1013)	R993T	[56]	-	3	-	Pathogenic
	R993S	[95]	-	1	-	Pathogenic
A998 (A1018)	A998I	[17]	-	3	-	Pathogenic
	A998V	[17, 56, 59, 60, 95]	-	9	-	Pathogenic
C1007 (C1027)	C1007R	[56]	-	1	-	Likely pathogenic
A1011 (A1031)	A1011T	[60]	-	1	-	Likely pathogenic
I1015 (I1035)	I1015S	[56]	-	1	-	Pathogenic
	I1015V	[60, 95]	-	2	-	Pathogenic
F1147 (F1167)	F1147C	[59]	-	1	-	Pathogenic
	F1147L	[59, 60]	-	2	-	Pathogenic
V1151 (V1171)	V1151F	[17, 56]	-	3	-	Pathogenic
I1152 (I1172)	I1152N	[17]	-	1	-	Likely pathogenic
V1153 (V1173)	V1153G	[87]	Type-2 [87]	1	-	Pathogenic
	Other gnomAD entries at this position:				V1153I (3×)	
R1183 (R1203)	R1183H	[60]	-	1	-	Likely pathogenic
	Other gnomAD entries at this position:				R1183C (2×)	
F1248 (F1268)	F1248L	[59, 60]	-	4	-	Pathogenic
D1273 (D1293)	D1273N	[60]	-	1	-	Likely pathogenic
P1336 (P1371)	P1336R	[3, 17, 59]	Type-3 [3]	4	-	Pathogenic
V1338 (V1373)	V1338M	[1, 17, 57, 59, 60, 73]	-	13	-	Pathogenic
I1352 (I1387)	I1352T	[59]	-	1	-	Likely pathogenic
M1354 (M1389)	M1354I	[3, 17]	No gating change [66]	2	-	Pathogenic/uncertain^#^
P1499 (P1534)	P1499L	[59]	-	1	-	Likely pathogenic
T1835 (T1879)	T1835I	[60]	-	1	-	Likely pathogenic
W1836 (W1880)	W1836X	[59]	-	1	-	Likely pathogenic

Criteria for the classification of the pathogenicity are given in Table 4. The reference *CACNA1D* sequence EU_363339 contains exon 8a but not exons 11, 32, 44 (gnomAD reference sequence NM_000720 contains exons 8b, 11, 32, and 44). If the residue position differs between the two sequences, the respective NM_000720 residue is given in parenthesis. *HOM*, homozygous; “X” indicates a STOP. *Monozygotic twins. *ns*, not specified; “-” indicates not measured (gating change) or not reported in gnomAD. ^#^M1354I fulfills the here described criteria of a pathogenic variant, but no typical gating changes have been observed in functional studies [66]. The W1836X variant lacks the distal C-terminal regulatory domain (DCRD), which disrupts an automodulatory interaction with the proximal C-terminus (PCRD), thereby interfering with channel gating (enhanced Ca^2+^-dependent inactivation and voltage sensitivity). The premature stop in the R510X variant likely results in a truncated, non-functional channel (loss of function)

## Molecular mechanism of altered channel gating in high-risk variants

As mentioned above all germline variants and the vast majority of APA/APCC variants occur in the voltage-sensing or pore-forming domain of the channel (Fig. 2).

**Fig. 3:**
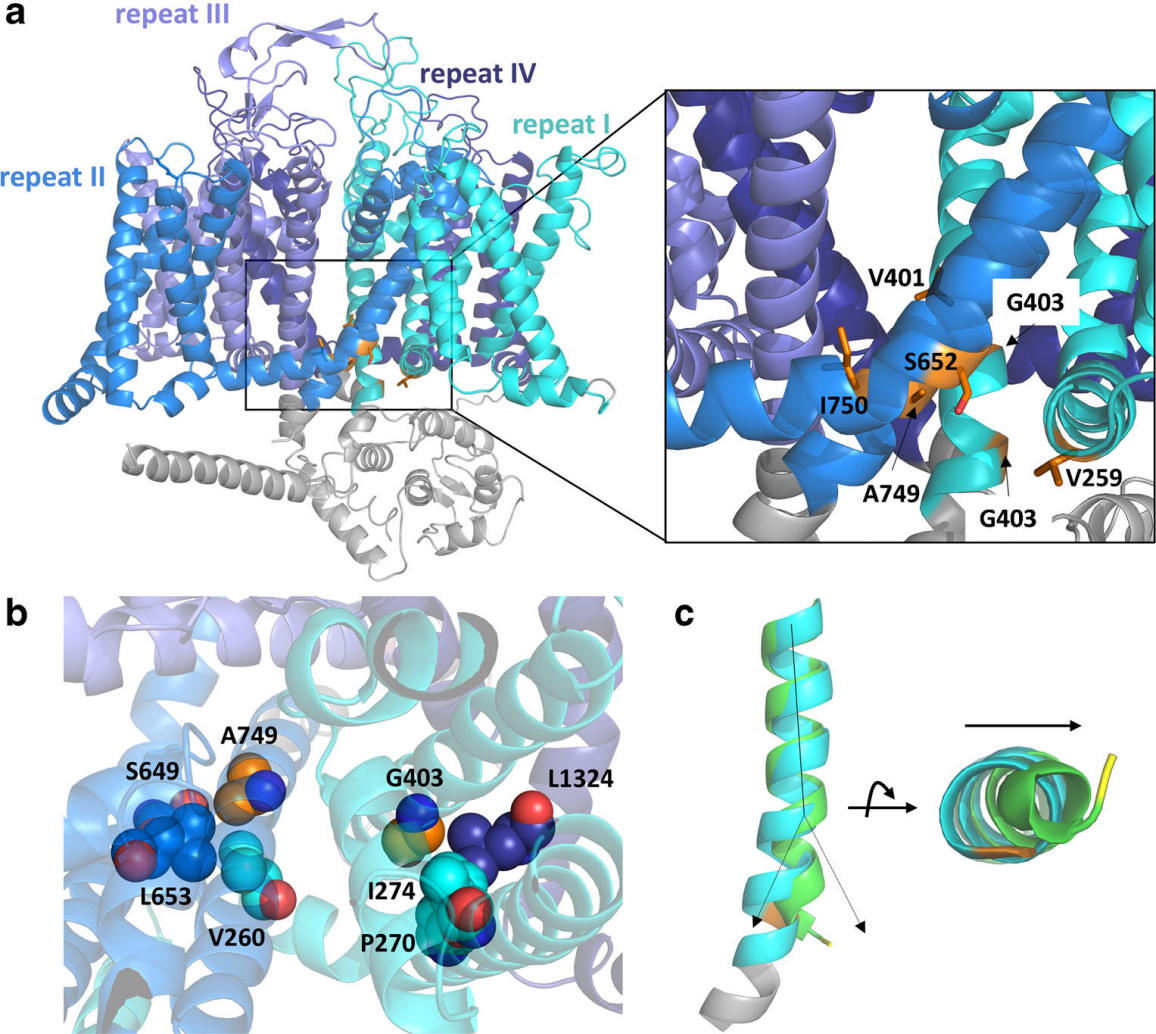
***CACNA1D*** structural models. (A) A homology model of the inactivated state based on rabbit Cav1.1 (PDB entry 5gjv, [93]). Transmembrane segments of homologous repeats I (cyan) and II (blue) are shown with germline de novo missense variants highlighted as orange sticks. They spatially cluster around the activation gate formed by S6-helices of repeats I and II. (B) The homology model of the *CACNA1D* resting state (based on PDB entry 6p6w, [92]) shows that A749 and G403 (orange spheres) tightly interact with surrounding residues on the S5 helix and S4-S5 linker (highlighted as spheres). Cartoon and carbon atoms depicted as spheres are colored according to the homologous repeats, whereas nitrogen and oxygen atoms of residues depicted as spheres are colored blue and red, respectively. (C) The pore-forming helix S6 requires conformational rearrangements of the backbone to facilitate pore opening in the activated state (green cartoon) compared with the resting state (cyan cartoon). G407 (S6 in repeat I) is highlighted in orange (resting state) and yellow (activated state)

We have used the SWISS-MODEL webserver [91] to generate a homology model of *CACNA1D* based on the apo structure of the inactivated rabbit Cav1.1 α1-subunit in complex with α2δ-, β-, and γ-subunits (PDB entry 5gjv, [93]). Mapping of the mutated residues included in Table 3 onto this structural model revealed spatial clustering around the activation gate of repeats I and II (Fig. 3A). In detail, V401, G403, G407, A749, and I750 are part of the pore-forming S6 segments, whereas V259 and S652 are located in close proximity on neighboring S4-S5 linkers (which connect the voltage-sensing domain (VSD) to the activation gate). These sections of the protein are critical determinants of multiple channel properties: V401 for example is part of a set of hydrophobic residues (V401, F747, F1147, and F1444) lining the pore. These residues form hydrophobic interactions when the pore is closed, thereby prohibiting Ca^2+^ conductance. Interestingly, variants of two other amino acids of this hydrophobic cluster—F747 and F1147—have been identified in APAs (Table 5). This suggests that disruption of these hydrophobic interactions generally alters channel function, independently of the exact residue affected, and it will be interesting to see whether a variant of the fourth residue in this cluster—F1444—will be observed in patients in the future.

G403 and A749 are part of the S6 G/A/G/A ring found at the intersection of the S4-S5 linker, S5, and S6 helices. They are involved in linker-S6 interactions and have been implicated in coupling of the VSD and the pore [23]. In a homology model of the resting state (based on the resting state of a BacNav channel, PDB entry 6p6w, [92]), these residues fit tightly in the intersection point (Fig. 3B). Therefore, any variant either increasing flexibility and diminishing hydrophobic contacts (i.e., alanine to glycine) or introducing larger and/or polar residues (i.e., alanine to threonine) is expected to interfere with, and thus destabilize, the modeled resting closed state conformation and promote open channel states.

In addition, A749, together with I750, is part of the “LAIA” motif that is highly conserved among L-type Ca^2+^ channels and mutation of any residues of the motif to proline resulted in altered activation properties in the related Cav1.2 channel [27]. Similar to the G/A/G/A ring, S652 and V259 are within another critical interaction site, connecting the S4-S5 linker of repeats I and II. Previous modeling studies suggested that loss of hydrogen bonds in the S652L variant could be responsible for the effect on channel function [26].

Finally, comparing the resting and activated (based on the active state structure of a BacNav channel, PDB entry 5vb8, [37]) state models of *CACNA1D* shows that G407 in the S6 segment is located within the pore region, where large movements of the backbone facilitate channel opening and closure (Fig. 3C). Please note that G407 corresponds to the last S6 residue that is still resolved in the template of the BacNav crystal structure, suggesting that downstream residues are subject to conformational flexibility.

Given that structural components regulating both VSD-pore coupling and channel activation as well as pore opening/closure are concentrated in regions surrounding the activation gate, it should not come as a surprise that residues within this area are particularly susceptible to structural changes and give rise to variants that alter the biophysical properties of the channel. We therefore propose that new variants in this region are likely to have a functional impact and should be characterized in detail.

**Fig. 4:**
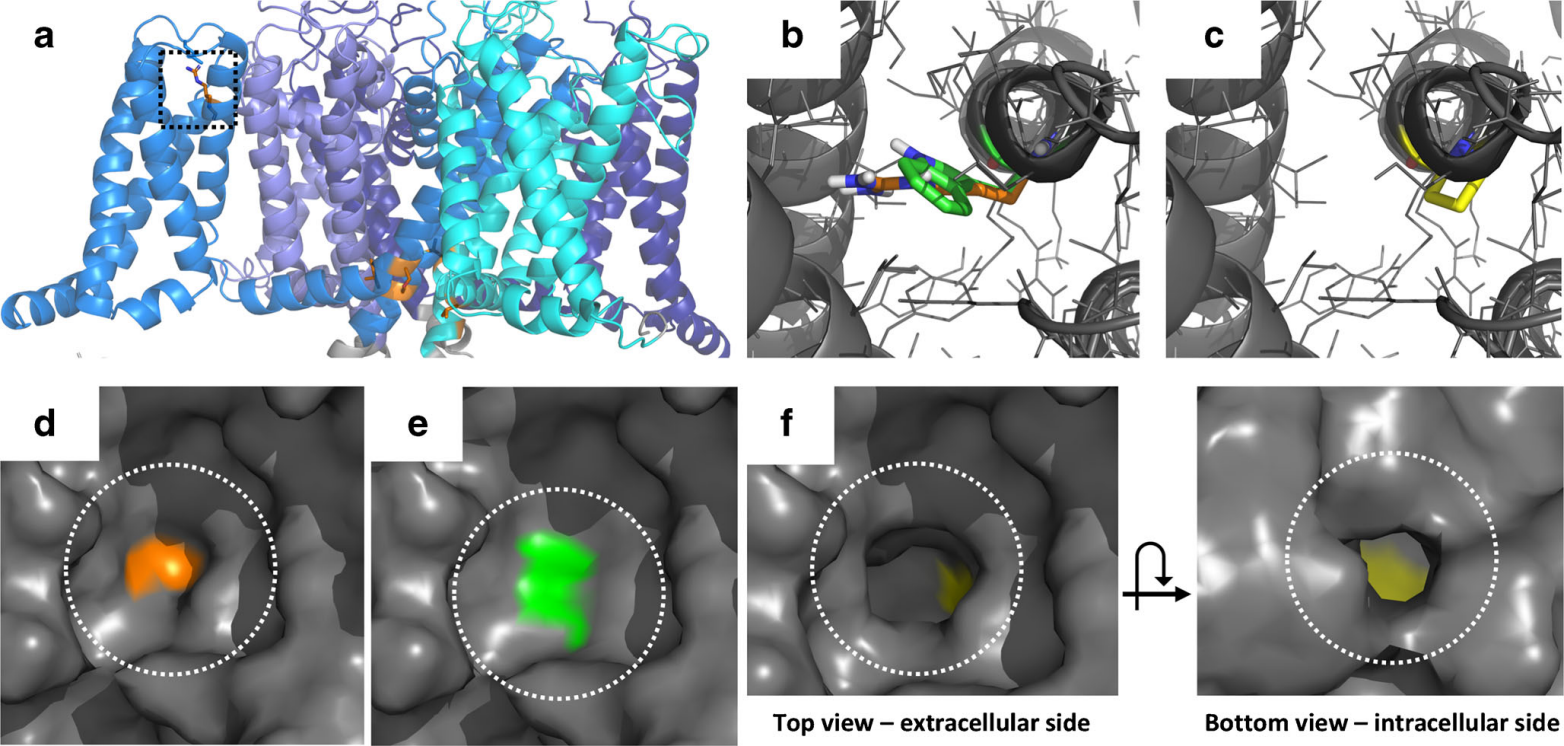
Modeling of the R619 variants. (A) R619 (orange sticks and highlighted by dotted lines) is the uppermost positively charged arginine in the VSD of repeat II. For reference, germline variants from Fig. 3A are shown as orange sticks as well. (B + C) Top view from the extracellular side onto the (B) wild-type R (orange sticks), and the W (green sticks) and (C) P (yellow sticks) variants in the Cav1.3 resting state model (gray). (D + E) In the resting state, both R619 (D, orange, highlighted by dotted lines) and the large hydrophobic side chain of the R619W variant (E, green, highlighted by dotted lines) provide a steric barrier that prevents ion leakage through the surface of the VSD (gray surface). (F) The R619P variant (yellow) lacks this large, hydrophobic sidechain, thus creating a tunnel via which ions can potentially pass through to the intracellular side. For comparison, this tunnel is depicted from both the top and bottom view

Molecular modeling can also provide evidence for variant R619P as a likely-pathogenic type 4 variant (Table 2). R619W and R619P were reported in APAs and APCCs (Table 5). However, R619W was also reported in a control subject in the gnomAD database (Table 5), casting doubt on its pathogenicity. R619 is the first positive charge in the VSD of repeat II (Fig. 4A). Both the wild-type R619 and the R619W variant provide a steric barrier that seals the VSD and prevents ion leakage through the surface of the VSD (Fig. 4B, D, E). Modeling of the R619P variant suggests that the smaller side chain fails to provide this seal and thus creates a tunnel (Fig. 4C, F). This allows for ion leakage through the VSD in the resting state and thus generates a so-called ω-current, which favors membrane depolarization. Please note that mutation of the uppermost arginine in the VSD has been described as a general mechanism to create gating pores in voltage-gated ion channels [53] and that the Cav1.3 R619P mutant directly corresponds to the Nav1.5 R808P mutant associated with Brugada syndrome (R809P in the rat; [30]). Altogether, this suggests that the Cav1.3 R619P variant also has a high probability of being pathogenic.

## Estimated prevalence of high-risk *CACNA1D* de novo variants in neurodevelopmental disorders

Two recently published germline de novo *CACNA1D* variants identified in small patient cohorts of ASD without (T1376M, [31]) or with epilepsy (V1447L, [44]) could suggest that the prevalence of such de novo *CACNA1D* variants associated with neurodevelopmental disorders is higher as currently deduced from NGS of large patient cohorts. Both variants are de novo and absent in healthy controls (gnomAD database); however, due to a lack of detailed clinical or functional data, they were not included in Table 3. According to the ACMG classification system [69], we consider the V1447L variant as likely pathogenic due to its location within a well-established functional domain (S6, repeat IV). Importantly, residue V1447 is highly conserved among all Cavs, lies adjacent to the S6 G/A/G/A ring of repeat IV, and corresponds to I750 in repeat II (discussed above), further strengthening the assumption that mutation of this residue can indeed interfere with normal Cav1.3 channel function. This is less clear for the T1376M variant which is located at the beginning of the S5-S6 pore loop of repeat IV, and a functional impact on channel gating cannot be presumed in absence of functional data.

So far only 6 de novo *CACNA1D* variants have been identified in large-scale genetic studies in cohorts of ASD (A749G, G407R [70], 11,986 individuals; T1376M [31], 59 individuals), ASD with epilepsy (V1447L, [44], 103 individuals), and severe developmental disorders (2 x S652L [15], 1133 individuals). In these cohorts, the occurrence of *CACNA1D* variants is relatively rare (together 1:2214); thus, they are often not identified as high-risk genetic variants (as happened for S652L in [15] but see [26]). Additionally, patients with pathogenic *CACNA1D* variants can be affected by endocrine and/or neurological symptoms (Table 3) and might therefore meet the exclusion criteria of such studies, further contributing to their underrepresentation. Since the majority of the known de novo *CACNA1D* germline variants have been published as case reports or were even unpublished (we are aware of at least two additional unpublished patients affected by one of the pathogenic variants in Table 3), this supports the notion that such *CACNA1D* variants are likely underreported in the literature.

## Summary and perspectives

As described in this review, we begin to understand the clinical disease spectrum associated with pathogenic de novo *CACNA1D* missense variants. Since the symptoms can only be explained by a channel gain-of-function, L-type Ca^2+^ channel blockers are a mechanism-based and still promising therapeutic option. However, current data predict that not all variants may be responsive to these drugs and it still remains unclear which of the many neurological and neuropsychiatric symptoms will respond to therapy. To learn more about potential drug effects, it is important to collect information about as many patients as possible which requires that clinicians and geneticists publish newly diagnosed patients as case reports or directly communicate them to members of the scientific community familiar with *CACNA1D*-associated diseases. But most importantly, all these efforts also depend on the support of parents and/or other caring family members and their willingness to share this information with the scientific community beyond clinical routine. We take this opportunity to thank many of the parents and physicians who helped to provide the clinical information for this review.

## Electronic supplementary material

Supplementary Fig. 1 **Somatic de novo*CACNA1D*variants found in APAs and APCCs**. The predicted pathogenicity of the variants (for details see Tables 4 and 5) is indicated by the following color code: Pathogenic (red), likely pathogenic (blue), likely benign (green), uncertain (orange). (JPG 265 kb)

## Data Availability

Not applicable
