# Supplementary figures and images for: De novo CACAN1D Ca2+ channelopathies: clinical phenotypes and molecular mechanism
Source: Pflugers Arch. 2020 Jun 24;472(7):755–73. doi: 10.1007/s00424-020-02418-w (PMC7351864; doi:10.1007/s00424-020-02418-w)

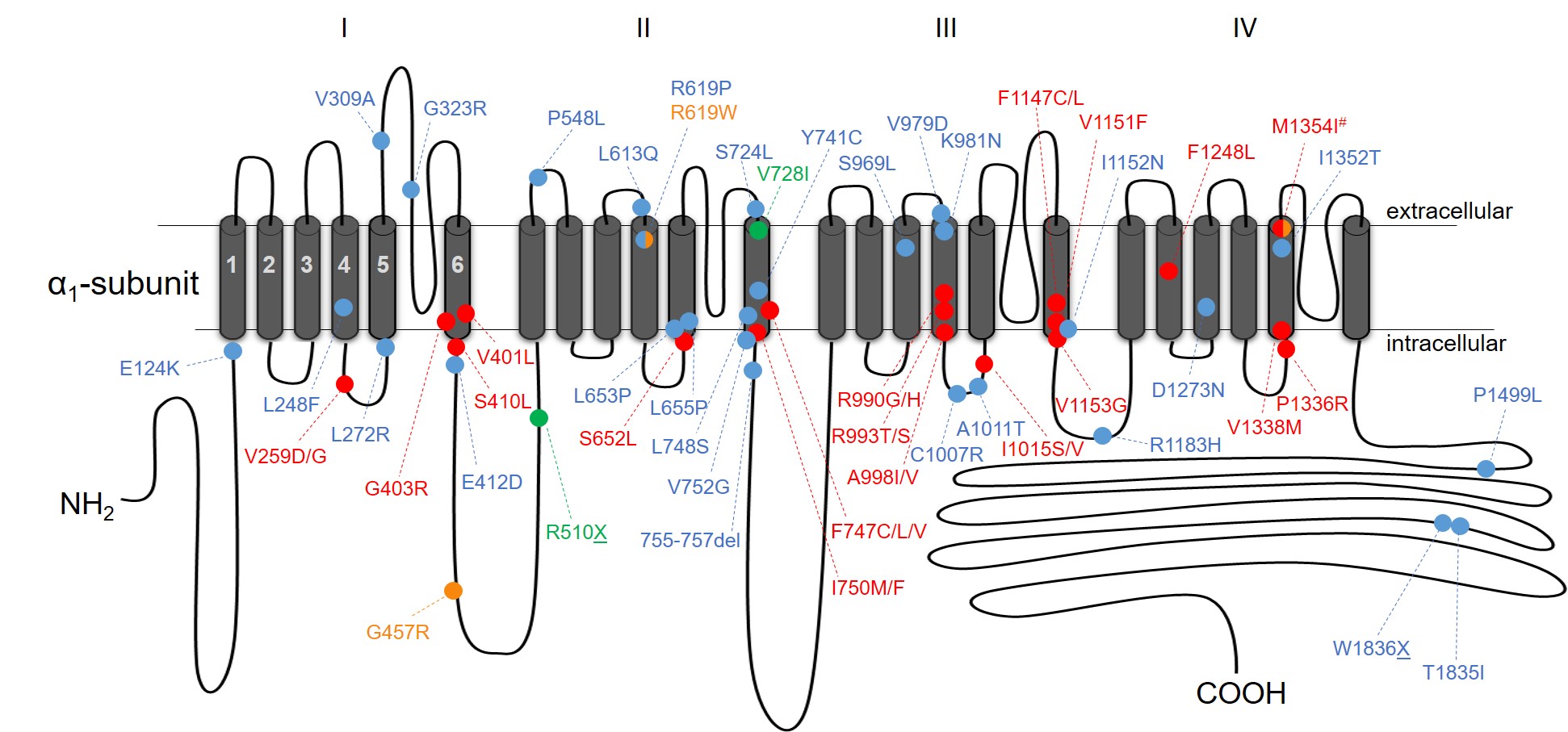

Supplement: Supplementary file 1 — Somatic de novoCACNA1Dvariants found in APAs and APCCs. The predicted pathogenicity of the variants (for details see Tables 4 and 5) is indicated by the following color code: Pathogenic (red), likely pathogenic (blue), likely benign (green), uncertain (orange). (JPG 265 kb) [file 424_2020_2418_MOESM1_ESM.jpg]
